# Nordic perspective on the International Consensus Meeting, Istanbul, May 8–10, 2025

**DOI:** 10.2340/17453674.2026.46432

**Published:** 2026-07-17

**Authors:** Ewout S Veltman, Mats Bue, P Koen Bos, Eerik Skyttä, Elin K Sober-Williams, Anna Stefánsdóttir, Marijn H Stelwagen, Justinas Stucinskas, Kaspar Tootsi, Marianne Westberg, Søren Overgaard

**Affiliations:** 1 Department of Orthopedics and Sports Medicine, Erasmus Medical Center, Rotterdam, Holland; 2 Department of Orthopaedic Surgery, Aarhus University Hospital, Denmark; 3 Department of Clinical Medicine, Aarhus University, Denmark; 4 Coxa Hospital for Joint Replacement, and Faculty of Medicine and Health Technology, Tampere University, Tampere, Finland; 5 Department of Orthopaedics, Institute of Clinical Medicine, University of Tartu, Estonia; 6 Department of Orthopedics, Skåne University Hospital, Lund, and Department of Clinical Sciences Lund, Division of Orthopedics, Lund University, Lund, Sweden; 7 Department of Orthopaedics and Traumatology, Lithuanian University of Health Sciences, Kaunas, Lithuania; 8 Department of Orthopaedics, Tartu University Hospital; Clinic of Orthopaedics, University of Tartu, Estonia; 9 Division of Orthopaedic Surgery, Oslo University Hospital, Oslo, Norway; 10 Copenhagen University Hospital Bispebjerg, Department of Orthopaedic Surgery and Traumatology and Department of Clinical Medicine, University of Copenhagen, Denmark

This perspective presents the conclusions and viewpoints of the Nordic Orthopaedic Foundation (NOF) on selected key questions (Table 1) from the third International Consensus Meeting (ICM) on orthopedic infections, held in May 2025 (https://www.icmortho.org/). The selection of questions for this article was determined through discussion among the authors and based on perceived interest within the orthopedic community in the NOF countries. Questions could be included for several reasons: to provide a specific Nordic perspective in contrast to the ICM recommendation, to highlight an ICM recommendation that could change current treatment, or to emphasize that the NOF perspective is in line with an ICM recommendation despite discussion of the topic in other regions.

**Table 1:** List of selected key questions

HK10: What is the definition of PJI?
Patient optimization G4: Does preoperative skin decolonization reduce the incidence of surgical site infections (SSI) in patients undergoing major orthopedic surgery? G5: Is there a role for universal screening for methicillin-resistant *Staphylococcus aureus* (MRSA) in patients undergoing major orthopedic procedures? G11: Should prophylactic antibiotics be altered for patients undergoing major orthopedic procedures based on their comorbidity profile? G13: What are the modifiable risk factors for postoperative infection in patients undergoing major orthopedic surgery? G14: Is preoperative anemia a risk factor for SSI/periprosthetic joint infection (PJI) for patients undergoing major orthopedic surgery? G20: Is there a role for nutritional supplementation for prevention of SSI in patients undergoing major orthopedic surgery?
Antibiotics G9: What is the optimal antibiotic prophylaxis for patients who have penicillin allergy? HK81: What is the optimal prophylactic antibiotic for patients undergoing primary arthroplasty? HK83: Is there a role for extended oral antibiotic prophylaxis for patients at high risk of infection after primary total joint arthroplasty HK84: Does extended antibiotic prophylaxis reduce PJI rate in patients undergoing aseptic hip or knee revision arthroplasty HK86: What is the optimal intravenous prophylactic antibiotic for patients undergoing assumed aseptic revision arthroplasty? HK88: Should prophylactic antibiotics be withheld during revision arthroplasty for aseptic failure until culture samples are taken? HK89: Should prophylactic antibiotics be altered for patients undergoing primary arthroplasty who have a history of prior PJI in another joint? G36: Does the use of vancomycin powder in the wound reduce the incidence of SSI/PJI in patients undergoing major orthopedic procedures? G78: Can oral antimicrobials be used for the treatment of implant-associated infection? G79: Is there a role for administration of rifampicin for patients undergoing surgical treatment for implant-associated infections? HK95: Does intraarticular antibiotic infusion have a role in the management of patients with PJI?
Diagnosis HK33: Is there a role for histopathological testing of joint tissue in the diagnosis of PJI? G72: Is there a role for sonication of implants obtained during revision surgery? HK35: Is there a role for the use of molecular techniques in isolation of infective organism(s) causing PJI?
Surgical management HK 48: What patients are candidates for debridement, implant retention, and antibiotic administration (DAIR)? HK59: Are there any absolute contraindications to performing 1-stage exchange arthroplasty for patients who have chronic PJI? HK60: During 1-stage exchange arthroplasty, should we use 2 separate instrument setups and re-drape after resection? HK68: Is there a role for abbreviated 2-stage exchange arthroplasty? HK75: Is there a role for 1.5-stage exchange arthroplasty in PJI? HK99: Is there a role for a 2-week antibiotic holiday in patients undergoing 2-stage exchange arthroplasty for PJI?
Epidemiology and registries G100: Is the epidemiology of orthopedic implant-associated infections changing? G101: Can registries be used to conduct research on orthopedic infections?

All documents can be found on PubMed using the following link: https://pubmed.ncbi.nlm.nih.gov/?term=(%22j%20arthroplasty%22%5BJournal%5D)%20AND%20(ICM%202025)&sort=&page=4

The questions have been divided into the following topics: patient optimization strategies, antibiotics, diagnosis, surgical management, and perspectives. For each question, the recommendation is copied directly from the ICM document, the rationale is a short summary of the original version, and the NOF perspective is written after discussion within the author group. Questions are marked with a letter and number combination; a prefix G states it is a general question, and a prefix HK states it is related to hip–knee pathology. For more detailed information on a specific question, we encourage readers to consult the full ICM document linked to that question.

Over 1,000 orthopedic surgeons, infectious disease specialists, microbiologists, and other professionals from more than 100 countries gathered for the meeting.

The introduction of the unified definition of periprosthetic joint infection (PJI), further discussed in HK10, was a notable highlight of the meeting.

Before the meeting, delegates submitted over 2,000 questions covering all aspects of orthopedic infections. 394 questions were selected by the ICM committee and addressed within specific domains, including general topics (102), hip and knee (102), shoulder (86), spine (72), and biofilm research (32). Each question was addressed through working groups, conducting thorough systematic reviews and meta-analyses. Each question required a recommendation and was presented orally (3 min), followed by a discussion among delegates and a formal voting process (% agree, % disagree, % abstain). The voting results were immediately presented.

Notably, countries NOF were strongly represented across all relevant specialties and included delegates from Norway, Sweden, Finland, Denmark, the Netherlands, Lithuania, and Estonia (Table 2). NOF, as an organization, was not formally involved in the ICM process.

**Table 2:** Participants from the Nordic Countries in alphabetical order

Physical attendance Denmark: Armita Abedi **^a,b,^** Mats Bue, Per Gundtoft, Louise Kruse Jensen, Jeppe Lange, Søren Overgaard **^a^** Estonia: Piret Mitt, Elin Kersti Sober-Willams, Kaspar Tootsi Finland: Meeri Honkanen, Kaisa Houtari, Eerik Skyttä Lithuania: Giedrius Kvedears, Augstinus Rimkunas, Alfredas Smailys, Justinas Stucinskas, Danguole Vaznaisiene The Netherlands: Koen Bos, Jan Geurts, Jon Goosen, Nanne Kort, Jesse Kuiper, Geert Meermans, Dirk Jan Moojen, Bart Pijls, Joris Ploegmakers, Denise Telgt, Karin Veerman, Ewout Veltman, Huub de Visser, Frank-Christiaan Wagenaar, Bart van der Wal, Marjan Wouthuyzen-Bakker, Erlangga Yusuf, Wierd Zijlstra Norway: Marianne Westberg, Tina Strømdal Wik Sweden: Maziar Mohaddes, Liliana Andrea Morales Laverde, Stergios Lazarinis, Ola Rolfson, Anna Stefánsdóttir, Staffan Tevell, Jonatan Tillander, Margarita Trobos, Annette W-Dahl
No meeting participation—involved in questions Denmark: Kirill Gromov, Per Kjaersgaard Andersen, Anders Odgaard, Marc Stegger The Netherlands: Chris Arts, Peter Croughs, Harmen Ettema, Jakob van Oldenrijk, Henk Scheper, Charles Vogely Sweden: Nils Hailer, Olof Thompson

a Hip and Knee Executive Committee.

b Academic Director.

The consensus process constitutes a well-established methodological framework for addressing questions characterized by limited evidence. However, both the process itself and the recommendations depend on the available evidence, which is why it highlighted several knowledge gaps in orthopedic infection management.

The quality of question significantly influenced the nature of the resulting recommendation. This sparked considerable debate, revealing differences in geographical interests. The number of delegates per country varied significantly and their level of expertise was not disclosed. The process was hindered by a lack of complete transparency, as individual registration and the number of votes were not disclosed.

## The unified periprosthetic joint infection (PJI) definition

### HK10: What is the definition of PJI?

The question on the definition of PJI was the only topic not voted on during the conference. A working group from several societies has done tremendous work to establish and agree on a unified definition. The involved societies are the European Bone and Joint Infection Society (EBJIS), the Musculoskeletal Infection Society (MSIS), the International Consensus Meeting (ICM), the Infectious Diseases Society of America (IDSA), and the European Society of Clinical Microbiology and Infectious Diseases (ESCMID). Infectious disease specialist Marjan Wouthuyzen-Bakker from Groningen presented the work, which, after approval by the steering committees of the involved societies, is expected to be published in the near future. According to the presentation standalone criteria for PJI are:

- Clinical (sinus tract).
- Microbiological (2 positive tissue cultures with a phenotypically indistinguishable pathogen, or 1 positive culture from synovial fluid or sonication fluid plus the same pathogen in 1 culture).
- Inflammatory markers and histology (synovial white blood count [WBC] > 3.0 x 10^9^/L, % polymorphonuclear leukocytes [PMN] > 75%, and ≥ 5 PMN/high-power field [HPF] in each of at least 5 HPFs).

Included among the supportive criteria are molecular tests, WBC scintigraphy, and fluorodeoxyglucose positron emission tomography and computed tomography (FDG-PET/CT; mostly for late presentations). No serological tests are included in the definition, reflecting their low sensitivity and their role as screening tools in the workup of a suspected PJI.

## Patient optimization strategies

Patient optimization strategies before, during, and after surgery to reduce infectious complications were evaluated. Elective arthroplasty provides the opportunity to optimize chronic disease management before surgery. Nevertheless, over-optimization and unnecessary delays before arthroplasty increase costs and harm and should be avoided.

### G4: Does preoperative skin decolonization reduce the incidence of surgical site infections (SSI) in patients undergoing major orthopedic surgery?[1]

*Response/recommendation:* Yes. The use of preoperative skin decolonization reduces the incidence of postoperative surgical site infections in patients undergoing major orthopedic surgery.

*Level of evidence:* Moderate.

*Rationale: Staphylococcus aureus* accounts for approximately 37% of SSIs, and 15–30% of patients are colonized with S. aureus on their skin. Concerns have been raised that these patients face a higher risk of developing PJI, which could be reduced through preoperative skin decolonization. A systematic review and meta-analysis demonstrated a reduced risk of any infection and of S. aureus infection following orthopedic surgery, specifically total joint arthroplasty. All strategies were cost-effective, with universal decolonization without screening being the most beneficial. The evidence for preoperative skin decolonization is not entirely consistent, as a few studies did not demonstrate a significant effect.

*Voting result:* Agree 79%, disagree 11%, abstain 10% (strong majority, moderate consensus)

*NOF perspective:* Preoperative skin decolonization appears to be a cost-effective treatment that reduces the risk of PJI. Practice varies in the Nordic countries. For example, in Sweden, preoperative skin decolonization is routinely performed for all hip and knee arthroplasty patients, whereas in other countries it is not performed for unscreened patients or for patients at all. This practice variation may be explained by practical and cost issues.

### G5: Is there a role for universal screening for MRSA in patients undergoing major orthopedic procedures? [1]

*Response/recommendation*: Currently, there is no evidence to support universal screening for methicillin-resistant S. aureus (MRSA) to infer any benefit in patients undergoing orthopedic procedures. Given the cost-effectiveness of modern decolonization protocols, we recommend universal nasal decolonization in all patients undergoing major orthopedic procedures, preferably using a non-antibiotic antiseptic agent.

*Level of evidence:* moderate.

Rationale: Nasal colonization with S. aureus increases the risk of SSI in orthopedic patients. Traditional strategies rely on screening for MRSA followed by targeted decolonization, but evidence for reducing SSI rates is inconsistent. A systematic review for the 2025 ICM meeting found no significant reduction in SSI with MRSA, though study designs, protocols, and populations included in the analysis varied greatly.

*Voting result:* Agree 72.7%, disagree 18.2%, abstain 9.1% (strong majority, moderate consensus).

*NOF perspective:* The incidence of MRSA in the Nordic countries has historically been low, but recent trends show a worrisome increase in some countries. As MRSA infections are much more difficult and costly to treat, it is important to enforce proven preventive measures. Thanks to vigorous screening and genotyping, there is a clear overview of MRSA strains and the associated population characteristics. Despite enforcing the “search and destroy” policy to prevent MRSA from becoming endemic, the incidence is rising. Even though the scientific evidence did not show benefit for universal MRSA screening, the workgroup issued a recommendation for using universal decolonization in all patients undergoing major orthopedic surgery. The European Society of Clinical Microbiology and Infectious Diseases (ESCMID) has issued a strong recommendation to decolonize both MRSA and MSSA carriers using mupirocin.

### G11: Should prophylactic antibiotics be altered for patients undergoing major orthopedic procedures based on their comorbidity profile? [2]

*Response/recommendation:* Although tailored antibiotic prophylaxis may be considered for high-risk populations, and based on regional microbial epidemiology, altering antibiotic prophylaxis based on the patient’s comorbidity profile does not seem to affect infection rates.

*Level of evidence:* Moderate.

*Rationale:* Although comorbidities such as diabetes, malnutrition, obesity, cardiovascular disease, kidney disease, smoking, and immunosuppression increase SSI risk, adjusting prophylaxis solely on these factors does not significantly reduce PJI rates. Standard cephalosporin regimens remain effective. Targeted strategies show benefit in select settings: e.g., adding teicoplanin for MRSA in hip fracture surgery. Gentamicin improved gram-negative coverage in pediatric spine surgery, and dual antibiotic-loaded cement reduced the risk of deep SSI in hip hemiarthroplasty. Overall, comorbidity-based changes alone are not warranted, but tailoring prophylaxis to local resistance patterns and high-risk scenarios may improve outcomes.

*Voting result:* Agree 79%, disagree 12.9%, abstain 8.1% (strong majority, moderate consensus).

*NOF perspective:* In light of the low prevalence of antibiotic-resistant bacteria in the NOF countries and the literature review provided by the 2025 ICM workgroup, there is insufficient rationale to alter the regular antibiotic prophylaxis recommendations.

### G13: What are the modifiable risk factors for postoperative infection in patients undergoing major orthopedic surgery? [3]

*Response/recommendation:* Evidence suggests that more than 25 modifiable risk factors are associated with an increased risk of SSI and PJI in patients undergoing major orthopedic surgery. Common modifiable risk factors include smoking, obesity, diabetes, anemia, and malnutrition, while others such as alcoholism, vitamin D deficiency, preoperative antibiotic prophylaxis, rheumatoid arthritis, corticosteroid use, anticoagulants, poor dental hygiene, HIV infection, urinary tract infection, surgical site preparation, operation room environment, operative time, depression, history of proton pump inhibitors use, opioids, frailty, and medical comorbidities like congestive heart failure and chronic pulmonary, renal, and liver diseases also contribute to infection risk.

*Level of evidence:* Strong.

*Rationale:* Strong evidence supports that preoperative patient optimization of several modifiable risk factors (especially smoking cessation, weight reduction, glycemic control, nutritional support, and anemia correction) can reduce the risk of SSI and PJI. Optimization of patients with comorbidities (such as renal failure, liver failure, congestive heart failure, chronic pulmonary diseases, and HIV) is important in reducing the risk of SSI.

*Voting result:* Agree 77.0%, disagree 13.8%, abstain 9.2% (strong majority, moderate consensus).

*NOF perspective:* Patients with specific comorbidities—such as uncontrolled diabetes, obesity, malnutrition, active smoking, and immunosuppression—are at increased risk of postoperative infections following major orthopedic surgery and these should be optimized preoperatively. The Nordic countries stand out for their systematic surveillance and management of chronic diseases, driven by successful universal healthcare policies that promote strong adherence to evidence-based national guidelines.

### G14: Is preoperative anemia a risk factor for SSI/PJI for patients undergoing major orthopedic surgery? [4]

*Response/recommendation:* Yes. Preoperative anemia is a risk factor for SSI and PJI in patients undergoing major orthopedic surgery.

*Level of evidence:* Moderate.

Rationale: Current evidence indicates that preoperative anemia is an important risk factor for SSI and PJI in major orthopedic surgery. Despite limitations in the available studies analyzed by the ICM 2025 workgroup, the findings highlight anemia as a modifiable factor that warrants careful optimization to improve infection prevention.

*Voting result:* Agree 91.4%, disagree 5.7%, abstain 2.9% (total consensus, unanimity).

*NOF perspective:* Current evidence indicates that preoperative anemia is a significant and modifiable risk factor for PJI in major orthopedic surgery, which should be carefully optimized to reduce postoperative risks.

### G20: Is there a role for nutritional supplementation for prevention of surgical site infection (SSI) in patients undergoing major orthopedic surgery? [5]

*Response/recommendation:* Protein-based nutritional supplementation reduces the occurrence of early SSI in major orthopedic surgery, particularly in patients who are of poor nutritional status.

*Level of evidence:* Limited.

*Rationale:* Hypoalbuminemia is a recognized risk factor for failure of surgical treatment of PJI. Nutritional supplementation is increasingly viewed as a preventive strategy against SSIs in orthopedic patients. Protein-based interventions, including immunonutrition, have shown significant reductions in postoperative infections and improved recovery in elderly patients with hip fractures. Evidence for other supplements, such as vitamin C, vitamin D and zinc, remains limited, with no clear reduction in infection rates demonstrated.

*Voting result:* Agree: 87%, disagree: 8%, abstain: 5% (super majority, strong consensus).

*NOF perspective:* Protein-based nutritional supplementation appears to reduce complications, especially in malnourished orthopedic patients. Nutritional assessment and correction should be incorporated into infection prevention protocols, although the effectiveness of supplementation varies with patient-specific factors.

## Antibiotics

Antibiotic prophylaxis and therapy are crucial for preventing and treating PJI. Regional differences in pathogen susceptibility to antibiotics lead to discussions of optimal antibiotic regimens for preventing and treating PJI. Northern European countries have a particular situation regarding the general antibiotic susceptibility of common pathogens causing PJI. The rates of infections caused by multi- and panresistant bacteria are low compared with other European and global regions, presumably due to a strict antibiotic stewardship. The type of antibiotic, route of administration, and duration are important factors to consider when optimizing treatment regimens. For clarity, we have divided this section into subsections on antibiotic prophylaxis and antibiotic treatment.

## Antibiotic prophylaxis

### G9: What is the optimal antibiotic prophylaxis for patients who have penicillin allergy? [2]

*Response/recommendation:* It is safe and appropriate to administer a first-generation cephalosporin (cefazolin) as first-line antibiotic prophylaxis in the operating room setting for patients who have a documented penicillin allergy.

*Level of evidence:* Strong.

*Rationale:* The selection of preoperative antibiotic prophylaxis in patients with a reported penicillin allergy can be complex. Although approximately 10% of the population reports a penicillin allergy, clinically relevant reactions are rare, and true allergy is confirmed in only 0.7% to 3%. Cross-reactivity between penicillins and cephalosporins is reported to be low. A systematic review found that cefazolin was well tolerated, even in patients with a history of severe allergic reactions.

*Voting result:* Agree 88.2%, disagree 11.8%, abstain 0% (super majority, strong consensus).

NOF perspective: Cephalosporins (cefazolin or cefuroxime) can be safely used as antibiotic prophylaxis in patients with reported penicillin allergy. Allergic reactions are rare, generally mild, and easily treatable. If anaphylaxis to cefazolin is reported, it can be investigated with an allergy test.

### HK81: What is the optimal prophylactic antibiotic for patients undergoing primary arthroplasty? (6)

*Response/recommendation:* Cephalosporins, particularly cefazolin, are strongly recommended as first-line prophylaxis for primary arthroplasty based on consistent high-quality evidence, significant reductions in infection risk, and minimal adverse effects.

*Level of evidence:* Strong.

*Rationale:* A systematic review demonstrated that the use of cephalosporins reduced surgical site infection (SSI) rates by 40% compared with non-cephalosporin antibiotics. A large Swedish registry study further reported that clindamycin was associated with a 50% increased risk of infection compared with cloxacillin. Weight-based dosing of cefazolin reduces infection rates. The 2021 American Association of Hip and Knee Surgeons Annual Symposium recommended the following dosing regimen: 1 g for < 60 kg, 2 g for 60–120 kg, and 3 g for > 120 kg. Multiple studies have investigated whether adding vancomycin to cefazolin improves efficacy compared with cefazolin monotherapy; no significant differences in SSI or PJI rates were observed.

*Voting result:* Agree 98.3%, disagree 0%, abstain 1.7% (super majority, strong consensus).

*NOF perspective:* Substantial evidence supports cefazolin as the primary choice for antibiotic prophylaxis in patients undergoing primary arthroplasty. Given the low incidence of infections caused by cefazolin-resistant bacteria, especially in NOF countries, there is no need for any additional prophylaxis. Cloxacillin historically remains first choice in Sweden and many centers in Denmark. It can still be a valid option but the timing of administration is important, and the short half-life has to be considered.

### HK83: Is there a role for extended oral antibiotic prophylaxis (EOAP) for patients at high risk of infection after primary total joint arthroplasty (TJA)? [6]

*Response/recommendation:* There is insufficient evidence to recommend the routine use of EOAP in high-risk patients following primary TJA.

*Level of evidence:* Limited.

*Rationale:* EOAP, beyond the conventional single or 24-h antibiotic prophylaxis, has been proposed as a strategy to reduce the risk of PJI in high-risk patients. EOAP use after total hip arthroplasty (THA) reportedly rose by 366% between 2010 and 2022. Evidence regarding the efficacy of EOAP is conflicting. Several retrospective studies reported significant reductions in PJI rates among high-risk patients receiving EOAP. Conversely, recent retrospective studies reported no significant differences in PJI rates between EOAP and control groups, and a higher 90-day PJI rate in the EOAP group, although limited by a lack of data on EOAP protocols and additional risk factors. Concerns regarding antibiotic resistance have also emerged, including resistance to sulfamethoxazole, trimethoprim, and erythromycin in coagulase-negative staphylococci from EOAP recipients, as well as more frequent gram-negative infections.

*Voting result:* Agree: 94.8%, disagree: 2.8%, abstain: 2.4% (total consensus, unanimity).

*NOF perspective:* Current evidence is insufficient to support routine EOAP use in high-risk primary TJA patients due to conflicting results, a lack of high-quality studies, variability in treatment protocols, limited follow-up durations, and concerns over promoting antibiotic resistance.

### HK84: Does extended antibiotic prophylaxis reduce PJI rate in patients undergoing aseptic hip or knee revision arthroplasty? [7]

*Response/recommendation:* No. There is no concrete evidence that administration of extended antibiotic prophylaxis reduces the rate of PJI in patients undergoing aseptic revision knee or hip arthroplasty.

*Level of evidence:* Limited.

*Rationale:* The benefit of extended postoperative antibiotic prophylaxis in aseptic revision arthroplasty remains controversial.

A literature review identified 8 studies that compared short-term (≤ 24 h) vs extended (> 24 h) antibiotic prophylaxis in aseptic revision knee or hip arthroplasty. None of the individual studies demonstrated a statistically significant difference in PJI rates between the short-term and extended antibiotic groups. A recent randomized controlled trial, which was not included in the review as it had not yet been published, found no difference in PJI rates between a single preoperative dose of intravenous antibiotics and a regimen of 5 days of extended oral antibiotics in patients undergoing aseptic revision surgery.

*Voting result:* Agree 90%, disagree 7%, abstain 3% (super majority, strong consensus).

*NOF perspective:* Currently, there is insufficient high-quality evidence to support extended antibiotic prophylaxis following aseptic revision knee or hip arthroplasty; therefore, prolonging antibiotic prophylaxis is discouraged.

### HK86: What is the optimal intravenous prophylactic antibiotic for patients undergoing assumed aseptic revision arthroplasty? [7]

*Response/recommendation:* Given the absence of comparative studies, the optimal prophylactic antibiotic for patients undergoing assumed aseptic revision arthroplasty should follow the current evidence for patients undergoing primary total joint arthroplasty, which remains a weight-based intravenous dose of a first- or second-generation cephalosporin. Targeted use of additional vancomycin may be considered, but the evidence is limited to a single retrospective review.

*Level of evidence:* Weak.

*Rationale:* A comprehensive literature review found no studies investigating the optimal choice of prophylactic antibiotics specifically for aseptic revision arthroplasty. The 2019 American Academy of Orthopaedic Surgeons clinical practice guideline recommends a first- or second-generation cephalosporin or a glycopeptide for antibiotic prophylaxis, but the evidence was of limited strength. Several studies have investigated optimal prophylactic antibiotic regimens for primary arthroplasty and reported significantly lower PJI rates with cefazolin compared with non-cefazolin antibiotics.

*Voting result:* Agree: 75.6%, disagree: 20.4%, abstain: 4.0% (strong majority, moderate consensus).

*NOF perspective:* In the absence of comparative studies for aseptic revision arthroplasty, prophylaxis should follow primary arthroplasty protocols.

### HK88: Should prophylactic antibiotics be withheld during revision arthroplasty for aseptic failure until culture samples are taken? [7]

*Response/recommendation:* No. Prophylactic antibiotics should not be withheld to obtain culture samples during revision arthroplasty for aseptic failure.

*Level of evidence:* Strong.

*Rationale:* Historically, concerns that prophylactic antibiotics might reduce culture yield led to hesitation in their preoperative use. However, recent evidence challenges this view and supports administering antibiotics prior to incision to achieve systemic distribution and potentially reduce the risk of PJI. A literature review identified 9 studies, all of which showed no significant difference in culture positivity between patients receiving prophylactic antibiotics before and after tissue sampling. 2 randomized controlled trials in patients with known PJI found that preoperative antibiotics did not affect intraoperative culture results. Furthermore, 2 retrospective reviews that specifically compared aseptic revision patients receiving antibiotics before incision vs after sampling found no significant difference in positive culture rates.

*Voting result:* Agree: 94.9%, disagree: 5.1%, abstain: 0% (unanimous, total consensus).

*NOF perspective:* Prophylactic antibiotics in revision arthroplasty should be administered before surgery.

### HK89: Should prophylactic antibiotics be altered for patients undergoing primary arthroplasty who have a history of prior PJI in another joint? [6]

*Response/recommendation:* Unknown. Although patients with a history of PJI are at a higher risk of developing another PJI, no studies address this question specifically. The benefits of altering/extending prophylactic antibiotics in this patient population need to be weighed against the potential risks.

*Level of evidence:* Limited.

*Rationale:* Although multiple studies confirmed prior PJI as a risk factor for subsequent infection, no studies directly addressed modifying prophylactic antibiotic regimens based on prior infection history or the organisms involved. In cases of recurrent PJI, infections were caused by the same organism as the initial infection, as well as by new pathogens, indicating that tailoring prophylaxis solely to the original organism may be insufficient.

*Voting result:* Agree 92%, disagree 5%, abstain 3% (total consensus, unanimity).

*NOF perspective:* Little evidence is available to support altering antibiotic prophylaxis in patients with a prior PJI in a different joint. If no signs of infection are present at the time of surgery, a standard antibiotic prophylaxis seems sufficient.

### G36: Does the use of vancomycin powder in the wound reduce the incidence of surgical site infection (SSI)/PJI in patients undergoing major orthopedic procedures? [2]

*Response/recommendation:* Due to insufficient high-level evidence, we recommend against the routine use of topical vancomycin powder to reduce SSI/ PJI in major orthopedic surgeries.

*Level of evidence:* Limited.

*Rationale:* A systematic review and meta-analysis were conducted to evaluate the effectiveness of topical vancomycin in reducing SSI and PJI in patients undergoing major orthopedic surgery. 13 randomized controlled trials (RCTa) comprising 5,041 patients were included. Only 1 study, involving discectomy patients, demonstrated a significant reduction in SSIs. Another study on tibial plateau and pilon fractures found a significant reduction in Gram-positive cocci infections but no decrease in overall SSI rate. The meta-analysis did not identify a statistically significant reduction in SSIs (risk ratio [RR] 0.78; 95% confidence interval [CI] 0.60–1.03), even when stratified by surgical type. Among the 4 studies reporting adverse events, 1 reported acute kidney injury and rash in the vancomycin group.

*Voting result:* Agree 89.1%, disagree 9.1%, abstain 1.8% (super majority, strong consensus).

*NOF perspective:* The use of topical vancomycin powder is not recommended, as no significant reduction in SSIs or PJIs was observed in major orthopedic surgery, despite a possible decrease in Gram-positive cocci infections.

## Antibiotic treatment

### G78: Can oral antimicrobials be used for the treatment of implant-associated infection? [8]

*Response/recommendation:* Yes, appropriately chosen oral antimicrobials can be effective for treatment of implant-associated infections.

*Level of evidence:* Moderate.

*Rationale:* An RCT demonstrated non-inferiority of switching to oral therapy within 1 week compared with prolonged intravenous (IV) treatment in patients with bone and joint infections. Similarly, an RCT found no difference between switching to oral antibiotics at 2 vs 6 weeks in patients with PJI. Several retrospective studies have confirmed these findings. An RCT on fracture-related infections demonstrated non-inferiority of oral therapy compared with IV therapy. A systematic review assessing the optimal duration of IV treatment after PJI likewise found that a short IV course was non-inferior to prolonged IV therapy. Concerns regarding oral antibiotics primarily relate to bioavailability. Agents with high oral absorption are preferred. Obesity and bariatric surgery may alter drug absorption.

*Voting result:* Agree 91.2%, disagree 3.9%, abstain 5.0% (total consensus, unanimity).

*NOF perspective:* Literature supports the switch to oral antibiotics for implant-associated infections when selected based on pathogen susceptibility, bioavailability, and individual patient characteristics. Treatment in NOF countries is often switched to oral antibiotics after an initial short 1- or 2-week IV course.

### G79: Is there a role for administration of rifampicin for patients undergoing surgical treatment for implant-associated infections? [8]

*Response/recommendation:* Unknown. Despite supportive animal data, clinical studies examining the efficacy of rifampicin for patients undergoing surgical treatment of implant-associated infections remain conflicting. Well-designed randomized trials are needed before a clear recommendation can be given.

*Level of evidence:* Moderate.

*Rationale:* Rifampicin is increasingly used as an adjunct, particularly for Gram-positive infections treated with debridement, antimicrobial therapy, and implant retention (DAIR). 2 RCTs have evaluated rifampicin in staphylococcal PJI. One reported a non-significantly higher cure rate with rifampicin than with ciprofloxacin monotherapy, with significance in the per-protocol analysis. The other found no difference when rifampicin was added to cloxacillin or vancomycin. Systematic reviews show mixed results. One found no association between rifampicin and treatment success across 13 observational studies, while another reported a pooled risk ratio of 1.10 (CI 1.00–1.22) for rifampicin effectiveness. Evidence on optimal dose, duration, and timing is limited. Some studies suggest that ≥ 14 days or ≥ 90 days may be beneficial, whereas others support short-term induction therapy.

*Voting result:* Agree 90%, disagree 9%, abstain 1% (super majority, strong consensus).

*NOF perspective:* Current evidence does not conclusively support the superior efficacy of rifampicin-containing regimens over alternatives. However, until strong evidence against its use becomes available, the current recommendation for the use of rifampin in staphylococcal infections should be followed. Cessation of rifampicin should be considered in any case of serious side effects.

### HK95: Does intra-articular antibiotic infusion have a role in the management of patients with PJI? [9]

*Response/recommendation:* Yes. Intra-articular antibiotic (IA) infusion may be a safe and effective adjunct for managing PJI, delivering high local antibiotic concentrations with minimal systemic toxicity. Given its favorable safety profile and efficacy across various surgical strategies, it may be considered an important option in the management of PJI.

*Level of evidence:* Moderate.

*Rationale:* The available evidence on IA infusion is predominantly composed of case series. In DAIR procedures, IA infusion has shown promising results for acute, culture-negative infections. In single-stage revisions, IA antibiotic infusion has been effective against chronic PJI, multidrug-resistant organisms, polymicrobial infections, and culture-negative cases, with reported control rates of 72.7–90.2%. In a 2-stage revision, a randomized controlled trial comparing a 7-day IA irrigation protocol with a conventional 6-week 2-stage exchange with an antibiotic-loaded spacer found no difference in adverse events between groups.

*Voting result:* Agree: 61.4%, disagree: 24.6%, abstain: 14.0% (simple majority, low/no consensus).

*NOF perspective:* The literature on intra-articular antibiotic infusion for the treatment of PJI is scarce and of low quality. Despite promising outcomes reported in the available studies, standardized protocols regarding antibiotic choice, dosing, and duration are lacking.

## Diagnosis

Accurate and timely diagnosis remains the cornerstone of successful management of PJI. With rapidly advancing diagnostic tools, from molecular techniques to sophisticated imaging, the ICM questions on this topic were particularly interesting and highlighted both progress and ongoing challenges. The section fostered consensus on best practices, updated recommendations, and introduced a new unified PJI definition

### HK33: Is there a role for histopathological testing of joint tissue in the diagnosis of PJI? [10]

*Response/recommendation:* Histopathological analysis of deep tissue samples can be useful as a confirmatory criterion for diagnosing PJI of the hip and knee. Although frozen sections showed slightly lower sensitivities with similar specificities compared with permanent sections, it can be supported as an intraoperative confirmatory criterion, especially when preoperative results are inconclusive.

*Level of evidence:* Strong.

*Rationale:* Multiple studies have demonstrated a strong correlation between the presence of PMN in periprosthetic tissue and PJI. A systematic review and meta-analysis showed a pooled sensitivity, specificity, and diagnostic odds ratio (DOR), and summarized area under the curve (sAUC) was calculated at 78.7% (CI 77.1–80.3), 96.1% (CI 95.3–96.8), 119 (CI 60–237), and 0.96 (SE 0.01), respectively. However, a limitation of most studies is the reference standard they utilized to diagnose PJI. Overall, good diagnostic accuracy was demonstrated regardless of the chosen PJI definition, suggesting that histopathological analysis is a reliable test method for diagnosing PJI and can be recommended as an intra- or postoperative confirmatory criterion

*Voting result:* Agree: 92.5%, disagree: 4.4%, abstain: 3.1% (total consensus/unanimity).

*NOF perspective:* The use of histopathological analysis of tissue samples varies in the Nordic countries, even though histopathology is a diagnostic criterion for PJI. Histopathology should be implemented as a diagnostic tool in those centers treating patients with PJI. Question HK34 discussed which thresholds should be used for neutrophils in histopathological sections. The recommendation, supported by moderate-level evidence, was a threshold of ≥ 5 PMN/HPF in each of at least 5 HPFs, and 94.2% of delegates agreed (total consensus/unanimity).

### G72: Is there a role for sonication of implants obtained during revision surgery? [11]

*Response/recommendation:* The use of sonication of implants retrieved during revision arthroplasty may present potential benefits in improving the detection of infective pathogens. However, further research is needed to validate the efficacy of this technique.

*Level of evidence:* Moderate.

*Rationale:* Sonication, which uses high-frequency sound waves to dislodge adhered pathogens from surfaces, has gained attention as a potential tool for improving the detection of pathogens on implants or cement spacers. While sonication has shown promise in improving diagnostic accuracy, its role in routine clinical practice remains unestablished. In a meta-analysis of 12 studies, the pooled sensitivity, specificity, positive likelihood ratio, and negative likelihood ratio for detecting PJI by using sonication fluid cultures (SFC) were 0.82 (CI 0.76–0.87), 0.94 (CI 0.85–0.98), 14.2 (CI 5.4–37.8), and 0.19 (CI 0.14–0.25), for joint arthroplasty. Moreover, some studies suggest variability in its effectiveness depending on the type of implant, the pathogens involved, the specimen utilized for sonication (implant versus spacer), and the methods used for sonication. Sonication in aseptic revisions appears to have lower clinical value, as the sensitivity and specificity for predicting future PJI are only moderate.

*Voting result*: 94.3% agree (total consensus, unanimity).

*NOF perspective:* Sonication requires equipment and training, and the method is not widely used in the Nordic countries. Including sonication as a routine diagnostic method is not warranted until its added value has been further established. The importance of an antibiotic-free interval before tissue sampling was not highlighted, but should be emphasized.

### HK35: Is there a role for the use of molecular techniques in isolation of infective organism(s) causing PJI? [12]

*Response/recommendation:* Yes. Molecular techniques are promising adjuncts to conventional methods for diagnosing PJI and isolating infective organisms. These techniques may be of particular benefit in culture-negative cases, when rapid pathogen identification is critical, when rare pathogens are suspected, or in high-risk patients, such as those with a history of recurrent PJI.

*Level of evidence:* Moderate.

*Rationale:* The diagnostic efficiency of next-generation sequencing (NGS) and polymerase chain reaction (PCR) was evaluated by extracting data from 50 studies. Some studies have found NGS to be more sensitive than conventional culture, whereas others have found the opposite. Several aspects of NGS are being studied, such as the type of sample (synovial fluid, sonication fluid, biopsy), setting (preoperative aspiration, intraoperative), and the effect of ongoing (or recently paused) antibiotic treatment. Several PCR techniques have been evaluated, with varying results. PCR has the potential to shorten the time to diagnosis, and, in general, the technique is highly specific.

*Voting result:* 92.5% agree (total consensus/unanimity).

*NOF perspective:* NGS is becoming less expensive and more widely available. Yet, the technique is not to be used as a routine method but may become an important diagnostic tool, especially in difficult cases where an infecting pathogen cannot be identified by conventional methods. 16S rDNA is a routine method, whereas the role of multiplex PCR is still unclear.

## Surgical management

Different surgical strategies are available for the treatment of PJI. Discussions at the ICM 2025 highlighted a paradigm shift toward greater acceptance of 1-stage exchange arthroplasty compared with historical treatment practices. Ongoing debates also focused on the optimal timing of the second stage in 2-stage revisions, as well as patient selection criteria for debridement, antibiotics, and implant retention (DAIR).

### HK 48: What patients are candidates for DAIR? [13]

*Response/recommendation:* In general, all patients with acute onset of infection and with a stable prosthesis are candidates for DAIR. However, the expected infection eradication rate greatly depends on several patient and infection characteristics.

The following patients are considered good candidates for DAIR:

- Infection within 6 weeks of the index arthroplasty.
- Infection with an onset symptom of less than 7 days.
- Well-fixed and stable implants.
- Exceptions may apply

*Level of evidence:* Strong.

*Rationale:* DAIR is most effective in carefully selected PJI patients, with outcomes strongly influenced by the timing of intervention and the host–pathogen–procedure triad. Success rates decline rapidly with prolonged symptom duration. Optimal results occur within 2–7 days, whereas poor outcomes are seen beyond 3–4 weeks of symptom onset. Each additional day of symptoms decreases the odds of success by 7.5%. The interval between the index surgery and DAIR is crucial: DAIR performed within 14 days of the index procedure yields lower reinfection rates (24.3%) compared with procedures performed between 46 and 90 days (45.8%). Delaying intervention beyond 6 weeks markedly increases failure rates. Certain host factors, such as advanced age, inflammatory arthritis, ischemic heart disease, chronic obstructive pulmonary disease, chronic kidney disease, morbid obesity, smoking, immunosuppression, sepsis/bacteremia, as well as elevated inflammatory markers (CRP, ESR), are associated with poorer outcomes. Risk tools (KLIC, CRIME80) and machine-learning models aid stratification but do not replace clinical judgment.

*Voting result:* Agree 76.5%, disagree 11.7%, abstain 11.7% (strong majority, moderate consensus).

*NOF perspective:* DAIR is widely utilized for the management of acute or late-acute PJI across NOF countries. Optimal outcomes depend on careful patient selection, taking into account factors such as symptom duration, interval from index surgery, host comorbidities, presence of sepsis or bacteremia, soft tissue condition, and inflammatory marker levels. DAIR should be performed in all patients with an acute onset of infection and well-fixed, stable implants, with early intervention prioritized and mobile parts exchanged whenever possible.

### HK59: Are there any absolute contraindications to performing 1-stage exchange arthroplasty for patients who have chronic PJI? [14]

*Response/recommendation:* No. We do not feel that there are any absolute contraindications to 1-stage exchange arthroplasty. However, relative contraindications may include signs of systemic sepsis, severely immunocompromised status, and extensive soft tissue defects that compromise primary wound closure.

*Level of evidence:* Moderate.

*Rationale:* One-stage exchange arthroplasty remains debated, though growing evidence shows infection control comparable with 2-stage revision, with benefits of lower morbidity, better function, and reduced costs. The 2018 International Consensus Meeting identified systemic sepsis, extensive comorbidities, resistant organisms, culture-negative infections, and poor soft tissue coverage as potential contraindications. The only consistently recognized relative contraindications are sepsis and inadequate soft tissue conditions. Culture-negative and resistant infections, when managed in specialized centers with comprehensive antibiotic strategies, have shown comparable infection-control rates to those of standard cases. Host health remains a critical prognostic factor, as immunocompromised or medically complex patients have lower rates of infection eradication.

*Voting result:* Agree 90.1%, disagree 7.6%, abstain 2.4% (total consensus, unanimity).

*NOF perspective:* The concept of 1-stage exchange arthroplasty is gaining broader acceptance in NOF countries as supporting evidence continues to grow regarding its efficacy in specialized centers. The decision to perform a 1-stage exchange should be based on an individualized assessment that considers patient status, microbiological profile, surgical expertise, and institutional resources.

### HK60: During 1-stage exchange arthroplasty, should we use 2 separate instrument setups and re-drape after resection? [14]

*Response/recommendation:* Yes. The use of 2 separate instrument setups and the use of new drapes after resection in the setting of debridement and implant retention procedures (DAIR), 2-stage exchange, and 1-stage exchange arthroplasty may decrease the risk of contamination and the possibility of infection recurrence. Further studies specific to 1-stage exchange are needed.

*Level of evidence:* Limited.

*Rationale:* Recurrence of PJI remains a major concern, yet the optimal intraoperative contamination-prevention strategy is unclear. Centers vary in their use of dual-instrument setups with re-draping and a single-setup approach. Contamination increases with operative time and tray exposure and is higher in septic than aseptic revisions. Repeated antisepsis before closure reduces the rate of superficial infection. Dual setups in 1-stage revision achieve infection control rates of 83–88%, and in DAIR, dual setup with re-draping significantly lowers recurrence compared with a single setup (48% vs 75%, P = 0.04). Despite added time and cost, re-prepping and dual setup remain minimal investments compared with the burden of reinfection.

*Voting result:* Agree 81.3%, disagree 16.3%, abstain 2.5% (super majority, strong consensus).

*NOF perspective*: Current evidence suggests that using 2 separate instrument setups and re-draping after resection may reduce contamination and reinfection risk.

### HK68: Is there a role for abbreviated 2-stage exchange arthroplasty? [15]

*Response/recommendation:* A number of studies demonstrate that early reimplantation provides similar outcomes to the traditional 2-stage exchange with a reimplantation window of 4 to 12 weeks. The role of high-dose local antibiotics in improving clinical outcomes and the criteria for selecting suitable candidates remain undefined.

*Level of evidence:* Limited.

*Rationale:* The optimal timing for 2-stage revision arthroplasty remains debated, with no universally accepted interval. Most studies suggest a 4–12-week window, though reported ranges vary widely, from days to over a year. A 2–4 week window can be classified as “short” and 6–8 weeks as “long,” while others recommend 4–6 weeks or around 9 weeks, underscoring the lack of consensus. Some studies show no outcome differences between short and long intervals, whereas higher reinfection rates are reported with very early (< 4 weeks) or delayed (> 11 weeks) reimplantation, suggesting a potential 4–11-week “optimal window”. Emerging approaches, including abbreviated protocols and antibiotic-eluting devices, may allow earlier reimplantation.

*Voting result:* Agree 86%, disagree 9.4%, abstain 4.7% (super majority, strong consensus).

*NOF perspective:* There is considerable variability in the reimplantation interval used for 2-stage exchange arthroplasty across NOF countries. Emerging evidence suggests that early reimplantation of 2–4 weeks may achieve outcomes comparable to the traditional long-interval approach.

### HK75: Is there a role for 1.5 stage exchange arthroplasty in PJI? [15]

*Response/recommendation*: The 1.5-stage revision does not show inferior results compared with the 2-stage technique, with the advantage of reducing the number of additional surgical procedures.

*Level of evidence:* Weak.

*Rationale:* The 1.5-stage revision—implantation of definitive components fixed with high-dose antibiotic cement as a long-term or “destination” prosthesis—has emerged as a hybrid between 1- and 2-stage revision strategies. For the knee, the construct generally includes a new femoral component and an all-polyethylene tibial insert, whereas in the hip, both the femoral and acetabular components are cemented. This strategy aims for indefinite implant retention, reducing or eliminating the need for a second-stage revision unless reinfection, functional decline, or mechanical failure arises. Recent studies and meta-analyses show infection control rates comparable to 2-stage revision, with lower morbidity, fewer complications, improved function, and potential cost benefits. Results appear most favorable in medically fragile patients or those with limited life expectancy. However, indications remain variable, long-term survivorship data are limited, and national registries currently lack a dedicated category for 1.5-stage revisions.

*Voting result:* Agree 72.6%, disagree 12.8%, abstain 14.7% (strong majority, moderate consensus).

*NOF perspective:* The 1.5-stage revision concept is relatively new within NOF countries and used infrequently—often as an unplanned outcome of an intended 2-stage procedure that is never completed, rather than a deliberately chosen strategy. Its wider adoption is limited by surgical expertise and lack of knowledge. In the hip, cementing both components as a temporary, often suboptimally cemented construct is technically demanding, making many cases functionally equivalent to a 1-stage revision. In the knee, implanting only an all-polyethylene tibial component after the first stage may be difficult due to implant removal/related bone defects. Although it may be a viable option in selected patients, true 1.5-stage revision remains uncommon across NOF centers, and its role requires further clarification.

### HK99: Is there a role for a 2-week antibiotic holiday in patients undergoing 2-stage exchange arthroplasty for PJI? [16]

*Response/recommendation:* There is no conclusive evidence that a 2-week antibiotic holiday improves the outcome of 2-stage exchange in patients who have a PJI. On the other hand, continuous therapy may result in better outcomes for some, such as immunocompromised patients [40].

*Level of evidence:* Limited.

*Rationale:* An “antibiotic holiday” before reimplantation in 2-stage PJI revision was first described by Insall et al. (1983) to help detect persistent infection, and a 2-week cessation period later became common practice. However, its value remains uncertain, as serological thresholds are unreliable, cultures have low sensitivity, and delaying reimplantation may worsen outcomes. A PRISMA-based meta-analysis of 6 retrospective cohort studies comparing antibiotic holiday vs continuous therapy showed mixed results: 3 studies found no difference, 2 favored an antibiotic holiday, and 1 favored continuous therapy. The pooled risk ratio was 0.77 (CI 0.40–1.46) with substantial heterogeneity (I² = 63%, prediction interval 0.13–4.44).

*Voting result:* Agree 90.7%, disagree 7.0%, abstain 2.3% (total consensus, unanimity).

*NOF perspective:* Current evidence is insufficient to demonstrate superiority of either continuous or interrupted antibiotic therapy. In patients with an uneventful postoperative course after the first stage (characterized by healed soft tissues, favorable inflammatory marker trends, and good antibiotic tolerance), an antibiotic holiday is unlikely to enhance microbiological yield or improve treatment outcomes, while potentially prolonging overall treatment duration and immobility. Conversely, a brief period of antibiotic discontinuation may be reasonable for selected patients experiencing antibiotic toxicity.

## Epidemiology and registries

The Nordic countries have a long tradition of national databases covering hip and knee arthroplasties. To monitor epidemiology and trends, sufficient completeness and high data quality are needed. Nordic registers show high completeness of primary operations, whereas revision surgery is lower, especially in cases revised due to PJI, which is also shared by other national registers.

### G100: Is the epidemiology of orthopedic implant-associated infections changing? [17]

*Response/recommendation:* Yes. The number and population incidence of PJI is rapidly increasing, driven by an increased number of arthroplasty operations, an increase in cumulative incidence of early infections (particularly in hips), as well as an increased prevalence of people living with joint replacements, who are at risk for late PJI.

*Level of evidence:* Low.

*Rationale:* A systematic review was undertaken, including publications reporting hip or knee PJI. Due to variation in reporting metrics, no quantitative synthesis was performed.

In conclusion, the cumulative incidence of early PJI (within 90 days and up to 1 year) may be increasing over time, particularly following hip arthroplasty. There is no consistent, comparable evidence following knee arthroplasty. The proportion of late-acute PJI as a total of all PJI may also be increasing.

*Voting result:* Yes 94%, no 4.0%, abstain 2.0%. (total consensus, unanimity).

*NOF perspective:* Based on studies in NOF countries, the risk of revision due to infection after primary THA almost doubled, from 2004–2018. However, during recent years, the PJI incidence in hip and knee arthroplasty seem to have stabilized around 0.5–2%, depending on register and their capture methods.

### G101: Can registries be used to conduct research on orthopedic infections? [17]

*Response/recommendation:* Registries can be used to conduct research on orthopedic infections. However, factors such as data completeness, validation processes, and the consistency of infection definition should be described when data from registries is used.

*Level of evidence:* Moderate.

*Rationale:* Registries may be a valuable tool for studying PJI. However, their effectiveness depends on data completeness, validation, and consistent definitions of infection. Studies show wide variation in registry accuracy. Some, such as the Danish Hip Registry, improved from 67% to over 90% completeness when linked to microbiology data, while others, such as the Dutch Registry, significantly underreported infections.

Validation methods, such as linking registry data with clinical records and microbiology databases, help correct underreporting and misclassification. These validation efforts improved accuracy but also highlighted discrepancies due to non-reporting and misclassification. Yet, inconsistent definitions—ranging from surgeon-reported diagnoses to the MSIS criteria—make comparisons difficult. Despite these challenges, registries offer strengths like large sample sizes and long-term follow-up. With improved validation and standardization, registries can be powerful tools for infection research.

*Voting result:* Agree 94%, disagree 2%, abstain 4%. (total consensus, unanimity).

*NOF perspective:* The NOF countries have a strong tradition of joint registries, and are known for high-quality, near-complete national coverage and standardized data collection. There are individual national registries in Denmark, Norway, Sweden, the Netherlands, and Finland, as well as collaborations through the Nordic Arthroplasty Register Association (NARA). The Nordic model shows how collaborative registry science can identify trends and outcomes even in rare complications like PJI, as in a 2023 study, which found a rising risk of revision for infection over time across all countries but has stabilized during the last 5 years. Further, the Danish Hip Arthroplasty Register has been at the forefront of using strong data-linkage with other national registries, and in particular with microbiology databases. Further improvement of the individual registries within the NOF countries to incorporate additional relevant factors is under continuous evaluation.

## NOF concluding future remarks

This NOF perspective on the 2025 International Consensus Meeting highlights both the strengths and inherent limitations of consensus-based guidance in orthopedic infections, particularly in areas with limited high-quality evidence. Overall, the findings support a shift towards personalized, evidence-informed, multidisciplinary decision-making rather than routine escalation of treatment, especially on antibiotic strategies, given ongoing concerns about antimicrobial resistance.

Emerging diagnostic modalities, including molecular techniques, are promising adjuncts but should complement, not replace, established methods and should be introduced wisely based on proven added diagnostic value. Surgically, a paradigm shift towards less invasive and more tailored approaches is evident, including wider acceptance of 1-stage revision, advanced types of spacers, and refined patient selection for DAIR.

Importantly, the consensus process underscores ongoing knowledge gaps, global practice variability, and the influence of regional epidemiology. The Nordic model, characterized by robust registries, low resistance rates, and structured healthcare systems, provides a strong foundation for implementation. We encourage national and NOF efforts to implement the suggested standard of treatment, as it may improve care for PJI patients. Future efforts should prioritize high-quality evidence generation, enhanced methodological transparency, and sustained international collaboration to optimize prevention and management strategies in orthopedic infections.
